# Potential Use of Propolis in Phytocosmetic as Phytotherapeutic Constituent

**DOI:** 10.3390/molecules27185833

**Published:** 2022-09-08

**Authors:** Narimane Segueni, Salah Akkal, Kadour Benlabed, Gema Nieto

**Affiliations:** 1 Laboratory of Natural Products and Organic Synthesis Campus Chaabat Ersas, Faculty of Science, Department of Chemistry, University Mentouri-Constantine 1, Constantine 25000, Algeria or or; 2 Faculty of Medicine, University Salah Boubnider Constantine 3, Constantine 25000, Algeria; 3 Unit of Recherche Valorisation of Natural Resources, Bioactive Molecules and Analyses Physicochemical and Biological (VARENBIOMOL), Faculty of Science, Department of Chemistry, University Mentouri-Constantine 1, Constantine 25000, Algeria; 4 Department of Food Technology, Food Science and Nutrition, Faculty of Veterinary Sciences, Regional Campus of International Excellence “Campus Mare Nostrum”, University of Murcia, 30071 Murcia, Spain

**Keywords:** propolis, phytocosmetics, antioxidant activity, antibacterial activity, wound healing activity

## Abstract

Phytocosmetic is an important aspect of traditional medicine in several cultures. Researchers are now focusing to find new and effective ingredients of natural origin. Propolis is a natural beehive product extensively used in traditional medicine. We aimed in the present study to investigate the potential use of propolis as an aesthetic and phytotherapeutic constituent in phytocosmetics. Propolis was extracted using 80% ethanol. Total phenolic and flavonoid contents were determined calorimetrically. Free radical scavenging ability and reducing capacity were evaluated using four assays and expressed as IC_50_ values. Antibacterial activity was evaluated by the determination of minimum inhibitory concentration (MIC) on 11 Gram-positive and Gram-negative bacteria. The wound healing activity of 30% ethanolic extract and propolis ointment was studied using excision wounds in the anterio-dorsal side of the rats. The phenolic acid composition of the tested propolis was investigated using UFLC/MS-MS analysis. The tested propolis was rich in phenolic and flavonoid content and demonstrated an interesting antibacterial and antioxidant activity. Wounds treated with propolis appear to display a lesser degree of inflammation. Chemical analysis led to the identification of 11 phenolics. Among them, five are considered as main compounds: Chlorogenic acid (48.79 ± 5.01 ng/mL), Gallic acid (44.25 ± 6.40 ng/mL), Rutin (21.12 ± 3.57 ng/mL), Caffeic acid (28.19 ± 4.95 ng/mL), and trans-cinnamic acid (20.10 ± 6.51 ng/mL). Our results indicated that propolis can not only be used as a cosmetic ingredient but also be used as a preventative and curative constituent, which might be used as a barrier when applied externally on infected and non-infected skin.

## 1. Introduction

Propolis is a resinous balsamic and sticky material, with various colors and a pleasant odor, which honeybees collect from a variety of plants. The word propolis, derived from Greek, means defense of the city. Propolis is a multifunctional material with numerous uses. Bees use propolis to cover holes and combs, restore damage, and strengthen and seal the hive’s borders and entrances [1,2]. In addition to its use as a building material, propolis is also used as a defense material protecting the colony against parasites, and infections [3]. Propolis’ chemical composition varies according to the plant that can be found in a specific region. The constituents of propolis are different, and vary according to climate, season, location, and year [1,2,3]. The most important pharmacologically active constituents in propolis are flavonoids, phenolics, and aromatics. Flavonoids are thought to account for much of the biological activity of propolis. Furthermore, investigations have shown that they exhibit spasmolytic (quercetin, kaempferol and pectolinargenin), anti-inflammatory (acacetin), anti-ulcerative (apigenin), or antimicrobial (pinocembrin and galangin) activities [4]. Aromatic acids and their esters have also been shown to have anti-fungal, anti-viral, and antibacterial properties [5].

Propolis has been used as a traditional remedy and complementary medicine in different cultures all over the world. Propolis was mainly used for sore throats and treating wounds, etc. [3]. Several studies have shown various biological activities of propolis including antibacterial, antifungal, antiviral, immunomodulating, antioxidant, anti-inflammatory, antiulcer, local anesthetic, hepatoprotective, and antitumor effects [6,7,8,9]. For a long time, research on propolis and its application were focused on dealing with human health. Propolis is considered a dietary supplement and is widely accepted and used by several countries to enhance health and prevent disease [10,11]. Nowadays, new emerging fields are growing. Propolis is used to improve the productivity and performance of livestock such as poultry, fish, Nile tilapia, etc. [12,13,14,15]. Propolis extracts, in particular, ethanol and water extracts, were reported to enhance the microbial durability and quality of foods during storage of meat, fruits, vegetables, milk, etc. In addition, the used extracts contribute to the chemical and physical properties of food [16]. Propolis extracts are also used in food packaging [17,18,19].

In the last recent years, consumer interest and demand for natural and healthy products have increased, leading to the development of healthy foods and beverages, products for medical devices, over-the-counter preparations, and phytocosmetics containing propolis [3]. Phytocosmetics are an important aspect of traditional medicine in Algeria with an annual growth of 12% [20]. Recently researchers are interested in natural products not only for their individual bioactive compounds but also for the use of their different crude extracts [21]. Propolis has attracted researchers’ interest and many investigations have indicated that it possesses a large spectrum of biological activity in particular antioxidant and antibacterial properties. In addition, its wound healing properties were also evaluated. However, few works were focused on its potential use as a multifunctional material in phytocosmetics. In addition, little is known regarding the potential applications of Algerian propolis. For this purpose, we aimed in the present study to investigate the potential use of Algerian propolis as an aesthetic and phytotherapeutic constituent in phytocosmetics. Antioxidant and antibacterial properties were tested and wound healing properties were evaluated.

## 2. Materials and Methods

### 2.1. Propolis Collection

Propolis samples were obtained from a local company in Benibelaid situated in the wilaya of Jijel, Algeria, with geographical coordinates of North latitude 36°51′34″ and East latitude 6°08′30″.

### 2.2. Preparation of Ethanolic Extract of Propolis (EEP)

To extract biologically active compounds, an Ethanolic Extract of Propolis (EEP) was prepared. Crude propolis was first stored at 4 °C and grounded to obtain a fine powder. Ethanol/water (80/20, *v/v*) was used as solvent with a propolis/solvent ration 1/10 (*w/v*). The extraction procedure was carried out using maceration. A total amount of 10 g of propolis was used. The mixture was left in the dark for 72 h at room temperature. Extraction procedures were performed in triplicate. The macerates were combined, filtered, and concentrated to dryness under reduced pressure at 40 °C using a rotatory evaporator (BUCHI R-210, Rotavapor. The extracted material was then frozen, crushed, and dissolved in 80% ethanol (ethanol/water (80/20, *v/v*)) at various concentrations [22].

### 2.3. Determination of Total Phenolic Content (TPC) and Total Flavonoid Content (TFC)

The TPC of EEP was determined by the Folin-Ciocalteu method [23]. EEP (100 µL) at a final concentration of 20 µg/mL was diluted with 900 µL of distilled water and mixed with 5 mL of reagent (Folin-Ciocalteu 0.2 N). After 4 min, 4 mL of saturated Na_2_CO_3_ (75 g/L) was added. Then, the mixture was held for 2 h in the dark and at room temperature. Finally, the absorbance was measured at 765 nm. Results were expressed as Gallic acid equivalent using a calibration curve (y = 6.4487x + 0.0185) with R^2^ value as 0.9991.

TFC of EEP was determined by the aluminum chloride colorimetric method [24]. EEP (250 µL) at a final concentration of 100 and 1000 µg/mL was diluted with 1.25 mL of distilled water and mixed with 75 µL of NaNO_2_. After 6 min, 150 µL of 10% AlCl_3_.6H_2_O solution was added. Finally, 0.5 mL of 1M NaOH was added after 5 min. Before measuring the absorbance at 510 nm, 275 μL of distilled water was added. Results were expressed as Quercetin equivalent using a calibration curve (y = 0.3885x + 0.0084) with R^2^ value as 0.9869.

### 2.4. Antioxidant Activity

Antioxidant activity was evaluated using four different assays: DPPH and ABTS radical scavenging activity and Ferric ion and cupric ion reducing antioxidant capacity.

#### 2.4.1. DPPH Assay

A total volume of 100 µL of EEP at a concentration ranging between 10 and 100 µg/mL was mixed with 2 mL of DPPH (in methanol solution 100 µM) and incubated for 30 min at 37 °C. The absorbance was then measured against a blank at 517 nm. Results are expressed as μM Trolox equivalent to 1 g of propolis extract [25].

#### 2.4.2. ABTS Assay

Similar to DPPH assay, a total volume of 100 µL of EEP at a concentration ranging between 10 and 100 µg/mL was mixed with 1 mL of ABTS, vortexed for 10 sec, and left for 1 min. Finally, the absorbance was determined at 734 nm. Results are also reported as μM Trolox equivalent antioxidant capacity by 1 g of propolis extract [26].

#### 2.4.3. FRAP Assay

First FRAP reagent was prepared with 1 mM 2,4,6-tripyridyl-2-tiazine (TPTZ) and 2 mM ferric chloride in 0.25 M sodium acetate. A volume of 900 µL of the prepared reagent was mixed with 100 µL of EEP (10–100 µg/mL) and incubated for 4 min. The absorbance was then determined at 593 nm. Results were reported as μM Trolox equivalent antioxidant capacity by 1 g of propolis extract [27].

#### 2.4.4. CUPRAC Assay

A total volume of 100 µL of EEP at a concentration ranging between 10 and 100 µg/mL was mixed with 1 mL CuCl_2_ solution (0.01 M), 1 mL distilled water, 1 mL ethanolic neocuproine solution (7.5 × 10^−3^ M), and 1 mL ammonium acetate buffer solution (1 M). The mixture was incubated for 30 min then the absorbance was determined at 450 nm. Results were expressed as μM Trolox equivalent antioxidant capacity by 1 g of propolis extract [28].

### 2.5. Antibacterial Activity

The antibacterial activity of EEP was investigated using the agar micro-dilution method.

#### 2.5.1. Bacterial Strains

A total of 11 Gram-positive and Gram-negative bacteria were tested in the present study. Three strains (standards strains) were from American Type Culture Collection (*S. aureus ATCC 25923*, *E. coli ATCC 25922*, and *P. aeruginosa ATCC 27853*, Reston, VA, USA). The other tested strains were isolated from clinical sources. The tested clinical strains were as follows: *S. aureus*, *E. coli*, *P. aeruginosa*, *K. pneumonia*, *P. mirabilis*, *β hemolytic*, *α hemolytic*, and non-hemolytic *streptococci.* All bacterial strains were obtained from the bacteriology laboratory of Ibn-badiss UHC-Constantine (Constantine, Algeria). Bacterial strains were cultivated in nutrient broth and incubated at 37 °C for 24 h then transferred (2–3 colonies) into a nutrient agar plate and incubated again in the same conditions.

#### 2.5.2. Micro-Dilution Method

Minimum Inhibitory Concentration (MIC) values were determined using the micro-dilution method according to the CLSI guidelines [29]. For each strain, an inoculum suspension was prepared using a fresh culture in the stationary phase as described in a previous study [30]. Bacterial inoculums were adjusted to 0.5 McFarland (1.5 × 10^8^ CFU/mL) in 0.9% sterile saline buffer. The serial dilutions of EEP were prepared in Muller–Hinton agar to obtain a final concentration of EEP propolis ranging from 10 to 1400 μg/mL. After the solidification of Muller–Hinton agar, Petri plates were inoculated with bacterial suspension and incubated at 37 °C for 18 h. Ethanol was used as a control. In brief, equal volume of Ethanol was used and added to the control plate using the same conditions. MIC value was recorded as the lowest EEP concentration inhibiting the visible bacterial growth.

### 2.6. Wound-Healing Activity

#### 2.6.1. Extraction and Propolis Ointment Preparation

Ethanolic extract of propolis was prepared and used for the evaluation of wound-healing activity. Propolis (9 g) was frozen at −20 °C, and ground in a chilled mortar. Then, the round powder was extracted with ethanol (30 mL of 80% ethanol) with continuous stirring for 7 days. The extract was filtered and concentrated in an evaporator under reduced pressure. A 10% propolis ointment was prepared from the propolis extract by mixing 1 g of propolis extract with 9 g of petroleum jelly for a total of 10 g. A 30% ethanolic extract of propolis was prepared by dissolving 3 g of propolis extract in 10 mL of 80% ethanol.

#### 2.6.2. Animals

The experiment was carried out on 16 female Wistar albino rats weighing between 97.2 and 189 g. The rats were obtained from Pasteur Institute (Algiers, Algeria). Animals were housed at 21 °C with day/night cycle of 12 h. They had free access to water and standard rodent feed. The rats were acclimated for 10 days. Our study was approved by the Committee on Ethics of Animal Experiments of (DGRSDT) Minister of Higher Education and Scientific Research Algeria, which supported our project (Code: D01N0 1UN 040 12018 0002).

#### 2.6.3. Double Incision Wound Assay

After successful anesthesia, the back of each rat was depilated using a commercial barber hair cutter and skin sterilization with providone (betadine) and rinsed with an ethanol swab. Two excision wounds were inflicted by cutting away approximately 100 mm^2^ full thickness of the skin of a predetermined area on the anterio-dorsal side of each rat. The rats were divided into 4 groups of 4 rats each. They were kept in an individual cage. For group I, wounds were treated with petroleum jelly. Group II was treated with propolis ointment. Group III was treated with 30% ethanolic extract, and Group IV was left untreated. All treatments were applied once every 24 h. Both petroleum jelly and the non-treated wounds served as a negative control. Treatments were applied daily in the morning for 18 consecutive days. All the rules of asepsis were respected. The application of the different treatments was carried out using a sterile swab renewed at each application and for each rat. Macroscopic observation of the different wounds was carried out before each new application. All wounds were left exposed. Throughout the period of our study, we controlled the following parameters: rat weight, animal behavior, appearance of redness, edema and pimple, appearance and disappearance of crust, and reduction in wound area.

Reduction in the wound area was expressed as % of the original wound area (% rate of contraction = [(wound area day 0-Wound area day N)/Wounds area day 0) × 100]. The percent of wound healing area was measured at the beginning of the experiments and 3, 6, 9, 12, 15, and 18 days after that [30,31]. All data are expressed in percentage ± standard error.

### 2.7. UFLC/MS-MS Analysis

Ethanolic extract of propolis investigated in the present study was analyzed by the method described by Gültekin-Özgüven et al., 2015, using a Shimadzu 20A series ultra-fast liquid chromatograph (UFLC, Shimadzu Corporation, Kyoto, Japan) coupled to MS detector with electrospray ion source (ESI) and a triple quadrupole analyzer (API-3200 QTRAP, AB Sciex, Framingham, MA, USA). Identification and quantification of phenolic acid composition were performed using standard phenolic acids, namely, *p*-Hydroxybenzoic acid, gallic acid, vanillic acid, syringic acid, sinapic acid, caffeic acid, trans-cinnamic acid, ferulic acid, *p*-coumaric acid, and chlorogenic acid, by comparison of retention time and peak area of the used standard and compounds detected in EEP. In addition, rutin was also used. Separation was performed on an Inertsustain C18 column (150 mm × 4.6 mm, 3 μm) with a guard column (4.0 × 10 mm × 2) using a gradient of mobile phase A and B (7.5 mM formic acid and acetonitrile). An increasing gradient of B starting from 5% up to 95% in 20 min was used. The column temperature was set to 40 °C. A volume of injection of 20 μL for each standard mixture was used with a flow rate of 0.5 mL min^−1^. [32]

### 2.8. Statistical Analysis

All experiments were performed in triplicates and results were expressed as mean ± standard deviation (SD). One-way analysis of variance (ANOVA) and T-test were used to determine the difference between the tested parameters using SPSS software (version 20.0, Chicago, IL, USA).

## 3. Results

### 3.1. Determination of Total Phenolic Content (TPC) and Total Flavonoid Content (TFC)

**Figure 1:**
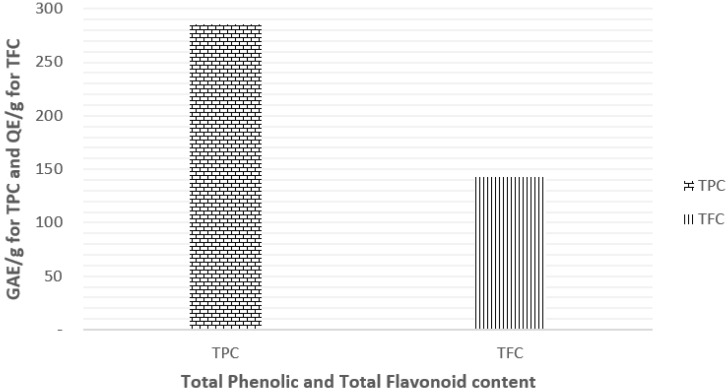
TPC and TFC of ethanolic extract of Algerian propolis. Note: The TPC and TFC were determined using Folin-Ciocalteu and aluminum chloride colorimetric methods. Results are reported as mean ± SD (*n* = 3) and expressed as GAE/g for TPC and QE/g for TFC.

The TPC and TFC of EEP were determined using Folin-Ciocalteu and aluminum chloride colorimetric methods and expressed as GAE (Gallic Acid Equivalent) and QE (Quercetin equivalent), respectively. Results are represented in Figure 1. The tested extract was found to be rich in both phenolic and flavonoid contents with a value of 285.69 ± 0.50 GAE/g for TPC and 143.05 ± 0.05 GAE/g for TFC.

### 3.2. Antioxidant Activity

**Figure 2:**
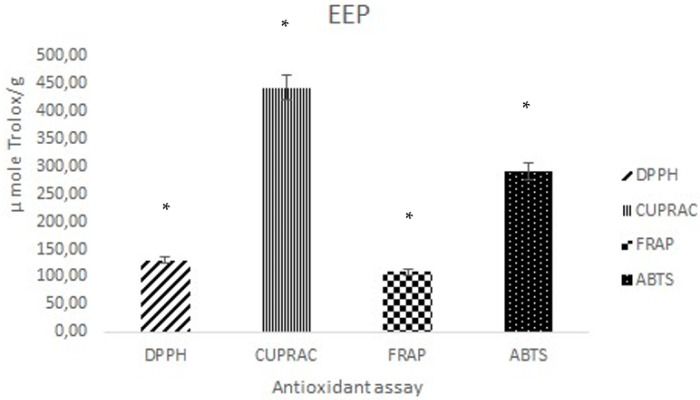
Antioxidant activity of ethanolic extract of Algerian propolis. Note: Results are reported as mean ± SD (*n* = 3) and expressed as μM Trolox equivalent antioxidant capacity by 1 g of propolis extract. Antioxidant activity was evaluated using four assays and the obtained results were compared. * = Significantly different value (*p* < 0.05).

In vitro antioxidant activity of EEP was evaluated using four different assays (DPPH, ABTS, FRAP, and CUPRAC). Results are reported as mean ± SD and are represented in Figure 2.

Our results indicated that EEP exhibited an interesting antioxidant activity with the highest value for CUPRAC assay (442.41 ± 22.35 μM Trolox/g) and the lowest value for FRAP assay (108 ± 24.05 μM Trolox/g). While DPPH and ABTS assays were 130.13 ± 5.43 μM Trolox/g and 290.40 ± 30.58 μM Trolox/g, respectively.

### 3.3. Antibacterial Activity

The antibacterial activity of EEP was evaluated by the micro-dilution method against 11 bacterial strains including standards and clinical strains. Results are summarized in Table 1.

For clinical strains, a first step was performed to evaluate their resistance to commonly used antibiotics. Several strains were found to be resistant. Resistance to antibiotics was as follows: For Gram-positive bacteria, two of the tested strains were resistant. *S. aureus* was resistant to β-lactamine and aminoside. While non-hemolytic streptococci was found to be resistant to erythromycin, thrimethoprime-sulfamethoxazol, and spriramycin. Regarding Gram-negative bacteria, three of the tested strains were resistant. *P. aeruginosa* was resistant to pefloxacin. *K. pneumonia* was resistant to amoxicillin associated with calvunilic acid. Finally, *P. mirabilis* was resistant to amoxicillin, pefloxacin, ticarcillin, nalidix acid, and thrimethoprime-sulfamethoxazol.

The micro-dilution method showed that EEP did not exhibit antibacterial activity against α hemolytic streptococcus. It is interesting to note that the tested extract exhibited a more pronounced activity on Gram-positive strains with an extremely low MIC value in particular against *S. aureus ATCC 25923* (0.06 ± 0.00 mg/mL). The clinical strains, which were found to be resistant to several antibiotics, were very sensitive to EEP with 0.20 ± 0.01 mg/mL for *S. aureus* and 0.60 ± 0.01 mg/mL for both *β hemolytic* and non-hemolytic *streptococcus.* Our results showed that EEP is less active on Gram-negative strains and on those for both standard and resistant clinical strains. MIC values ranged from 1.00 ± 0.00 mg/mL for *E. coli* (standard and clinical strain) to 1.20 ± 0.02 mg/mL for *P. mirabilis* and *K. pneumonia.*

### 3.4. Wound-Healing Activity

The wound-healing properties of EEP were investigated using the double incision wound assay. During the time of the experiment, the animals did not show any signs of excitation and did not react defensively. In addition, no mortality was seen in the animals during the study. Macroscopic observations of the healing process in the experimental (treated) and control group are summarized in Table 2.

Wounds treated with petroleum jelly and untreated wounds appeared to display a greater degree of inflammation as notable by the clinical signs of the inflammatory process: heat, redness, and swelling, which appear to be lessened in wounds treated with 30% EEP and propolis ointment. In the case of apitherapeutic treatment, the differences in wound appearance and animal behavior were visible after 48 h. Wounds were wet. Edema and reddening at the wound surroundings were smaller. On the 9th day of the experiment, the wound surface became visually smaller. The scabs started to come off at the boundaries of the wound surface, and a light-pink scar was visible underneath. This suggested an advanced epidermalization process. The applied apitherapeutic has a positive impact on the general health condition of the animals.

**Figure 3:**
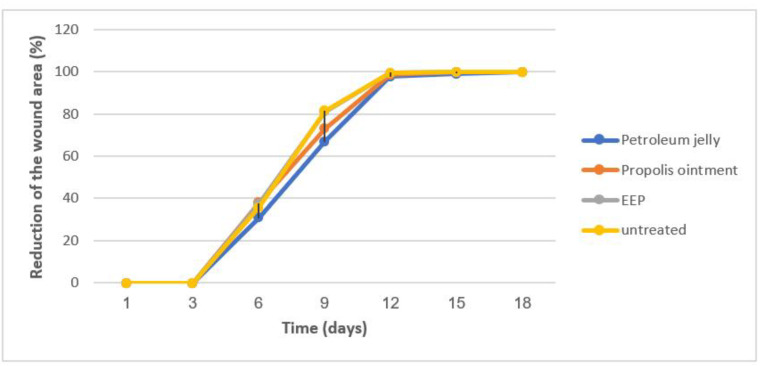
Wound-healing activity of ethanolic extract of Algerian propolis. The wound contraction of treated and untreated rats was expressed as a % of the reduction in the wound area. There was no statistically significant difference between treatment and negative control animals on most of the days.

The wound contraction of treated rats was evaluated and compared to the control. The area of the wound was measured at the beginning of the experiment and 3, 6, 9, 12, 15, and 18 days after that (Figure 3). There was no statistically significant difference between treatment and negative control animals on most of the days.

### 3.5. UFLC/MS-MS Analysis

The phenolic acid composition of the studied propolis was determined using UFLC/MS-MS analysis. Results are summarized in Table 3.

UFLC/MS-MS analysis detected the presence of 11 phenolic acids. Among them, five are considered as main compounds: Chlorogenic acid (48.79 ± 5.01 ng/mL), Gallic acid (44.25 ± 6.40 ng/mL), Rutin (21.12 ± 3.57 ng/mL), Caffeic acid (28.19 ± 4.95 ng/mL), and trans-cinnamic acid (20.10 ± 6.51 ng/mL).

## 4. Discussion

Natural ingredients have been used for centuries for skin care purposes [33]. In the last recent decade, an increasing interest in their consumption was observed. “Green consumers” are growing all over the world, in particular, in European countries. Consumer attraction has enhanced the development, research, and production of natural cosmetics. Natural and certified cosmetics are those composed of natural raw materials (certified or not) in their formulation. Natural raw materials might be vegetables, minerals, etc. [34].

Propolis is a resinous and balsamic beehive material used extensively since ancient times [21]. Propolis’s antioxidant and antibacterial activities are well documented. In addition, propolis has demonstrated a promising role in wound healing [35,36]. However, little is known about Algerian propolis. Therefore, we aimed in the present study to investigate the potential use of propolis as an aesthetic and its phytotherapeutic constituents in phytocosmetics.

To access the release of bioactive compounds, extraction with a suitable solvent must be performed. Ethanol was described as the best solvent for propolis extraction [37,38,39,40,41]. Several factors can affect propolis extraction such as the used solvent, the used method, and propolis botanical origin [42,43,44]. In addition, other factors such as time of application, temperature range, and PH are also described [45]. However, normal extraction (maceration) is still regarded as the most used and effective method for the extraction of propolis phenolic constituents [39]. Based on our previous investigation on Algerian propolis, ethanol 80% in the ratio of 1/10 was used in the present study. The chosen parameters led to the extraction of the highest amount of TPC and TFC with the highest antioxidant and antibacterial properties [22]. The use of ethanol 80% and its effectiveness is consistent with previously published data [45,46,47,48,49,50]. In a recent study, Kara et al., 2022, determined the percentage and ratio of ethanol used as a solvent in normal and ultrasonic extraction of propolis that led to the highest TPC and TFC and antioxidant activity. The 1/5 and 1/10 ratios were found to be the most suitable solvent ratios for minimum cost and maximum efficiency [45]. The use of ratio 1/10 is consistent with our previous investigation on Algerian propolis.

Total phenolic and flavonoid contents were determined calorimetrically. The tested extract was found to be rich in both phenolic and flavonoid contents with a value of 285.69 ± 0.50 GAE/g for TPC and 143.05 ± 0.05 GAE/g for TFC. TFC reported in the present study are lower than those reported for Algerian propolis collected from Laghouat [50], and Skikda and Oum El Bouaghi [51]. While TFC is in the same range as propolis collected from Constantine, and higher than propolis collected from Skikda and Oum El Bouaghi [50]. The reported amounts (TPC and TFC) are in the same range as Turkish propolis [45] and Brazilian propolis [49], and higher than those reported for Indonesian [39], Palestinian, Moroccan [52], and Romanian propolis [53,54].

The skin plays an important role in body protection against biological, chemical, and physical agents. Because of its function, the skin is under constant oxidative stress. Natural or synthetic active ingredients can be used in the formulation of topical preparation applied to the skin. The release of such bioactive constituents in the skin can help to better protect it [21,55]. This protection varied according to the pharmacological properties of those bioactive constituents. We reported, in the present study, our investigation of the antioxidant, antibacterial, and wound-healing activities of Algerian propolis. The studied activities can be useful for topic formulation.

Our results regarding antioxidant activity indicated that EEP exhibited interesting antioxidant activity with the highest value for CUPRAC assay (442.41 ± 22.35 μM Trolox/g) and the lowest value for FRAP assay (108. ± 24.05 μM Trolox/g). While DPPH and ABTS assay were 130.13 ± 5.43 μM Trolox/g and 290.40 ± 30.58 μM Trolox/, respectively. The results of the present study are higher than our previous investigation on Algerian propolis [22]. The tested propolis showed a strong scavenging activity against DPPH and ABTS radicals. The obtained values are in the same range as those reported for Colombian propolis [56], some Brazilian propolis [57], and Tunisian propolis [58]. Our results are lower than Turkish [59,60] and Brazilian [61,62] propolis and higher than some propolis collected from Argentine and Brazil [63,64]. Regarding their reducing capacities, the CUPRAC assay showed the highest capacity values compared to the FRAP assay. This is mainly due to the fact that copper has faster reaction kinetics [59]. Compared to other propolis, Algerian propolis showed higher reducing capacities than Brazilian propolis [65] and lower capacities compared to Indian [66] and Turkish propolis [59]. Based on the result concerning the antioxidant activity of Algerian propolis, EEP investigated in the present study could be used in the preparation of topical formulations to protect, prevent, and treat skin diseases involving oxidative stress. Propolis phenolic components, mainly polyphenols and flavonoids, have been widely investigated. Different propolis extracts and isolated compounds have been evaluated for their antioxidant properties [49,52,53,57]. However, only a few reports have focused on its antioxidant biological responses in topical formulation [21].

Propolis is a natural antimicrobial agent used as a multifunctional material by honeybees [1]. The antibacterial activity of propolis is one of the most documented pharmacological properties in the literature [52,67,68]. We investigated, in the present study, the antibacterial activity of EEP of Algerian propolis against 11 bacterial strains including standards and clinical strains (sensitive and resistant). Our results indicated that the tested propolis possesses a more pronounced activity on Gram-positive strains with the lowest MIC values for *S. aureus ATCC 25923*. This finding is in agreement with previous studies on Algerian propolis [22,50,51,69]. Several authors described a high antibacterial activity against Gram-positive bacteria and a limited activity against Gram-negative bacteria [52,67,68]. MIC values obtained for *S. aureus ATCC 25923* in the present study are in the same range as propolis collected from Lagouat located in the Algerian Sahara Atlas [50] and higher than four different propolis collected in the northeast of Algeria. The tested propolis showed MIC values ranging from 0.125 to 0.25 µg/mL and is more effective against *E. coli ATCC 25923*, *P. aeruginosa ATCC 15442*, and *K. pneumonia ATCC 43816* with MIC values ranging from 128 to 256 µg/mL [51]. Our propolis is more active than Turkish propolis against *S. aureus*, *E. coli*, and *P. aeruginosa* standard strains, which were found to have MIC values of 6.25 mg/mL, 6.25 mg/mL, and >25 mg/mL, respectively. Regarding resistant strains, our propolis is more active than Moroccan and Palestinian propolis against *S. aureus* resistant to vancomycin (MIC = 0.17–1.25 mg/mL). The tested clinical strain in the present study was more resistant (β-lactamine and aminoside) and exhibited a lower MIC value (0.20 ± 0.01 mg/mL). The *P. aeruginosa*-resistant strain exhibited a close MIC value to Moroccan and Palestinian propolis (0.625–1.25 mg/mL). While our propolis exhibited a lower MIC value for the *E. coli* resistant strain. It is important to note that the tested strain for Moroccan and Palestinian propolis was more resistant (resistance to Cefuroxime, Ceftriaxone, Cefaclor, Amoxicillin, Ceftazidine, Cefotaxime, Cephalothin, and Ciprofloxacine) [52]. In light of the result of the present section, Algerian propolis was found to possess an interesting antibacterial activity and can be not only useful but effective in the prevention and treatment of infections involving the studied strains.

Wound healing is a complex physiological process occurring as a response to injury [70]. Topical formulations with natural ingredients have been developed to accelerate this process. We investigated in the present study the wound healing properties of 30% propolis and propolis ointment in a double-incision wound assay. The macroscopic observation suggested a potential anti-inflammatory effect, as the reduction in different signs of inflammation (heat, redness, and swelling) were visible after 48 h. However, no statistically significant difference was observed on the wound contraction of treated and control animals on most days. Our results are in contradiction with those reported in the literature. The ethanol extract of Indian propolis was found to possess significant pro-healing activity. Propolis seems to accelerate various phases of tissue repair [70]. Similar results were reported in dogs. Propolis treatment significantly reduced the wound surface after 14 and 21 days. Wound contraction and re-epithelization were faster in the group treated with propolis [71]. Further investigations are in need to help to better understand the wound healing properties of Algerian propolis.

UFLC/MS-MS analysis detected the presence of 11 phenolic acids. Among them, five are considered as main compounds: Chlorogenic acid (48.79 ± 5.01 ng/mL), Gallic acid (44.25 ± 6.40 ng/mL), Rutin (21.12 ± 3.57 ng/mL), Caffeic acid (28.19 ± 4.95 ng/mL), and trans-cinnamic acid (20.10 ± 6.51 ng/mL). Phenolic compounds are described as direct antioxidants. Their activity is due to the reactivity of phenol moiety and hydroxyl substitution and their position in the aromatic ring. Different mechanisms of action have been noted. However, radical scavenging via hydrogen atom donation is described as the main mechanism. Phenolics act also as indirect antioxidants. They induce endogenous enzymes involved in the protection mechanism and act positively in the regulation of signaling pathways [72,73]. Phenolic acids are known to exhibit antimicrobial activities. The potential antimicrobial properties depend on the chemical structure. The saturation, length, number, and position in the benzene ring are of great interest [73,74]. PKa and lipophilicity are the key factors of phenolic acid solubility in the microbial membrane. They are determining factors in the diffusion pattern across the membrane and cause microbial cell cytoplasm acidification leading to cell death [75].

Wound healing is a complex process involving a series of cellular and molecular events that act to repair the defect in tissue integrity. Natural products such as phenolics were described to act by several mechanisms. They cause acceleration of the wound healing processes through their antioxidant, antimicrobial, and anti-inflammatory properties [76]. Among the identified phenolics in EEP of the present study, gallic acid [77,78], vanillic acid [79], caffeic acid [80], *p*-coumaric acid [81], and ferulic acid [82] were demonstrated to possess the described properties. In addition, they were described to accelerate wound contraction and reduce the epithelization period [76]. Rutin was also found to reduce wound area and increase lesion closure through increasing the production of lipid peroxidative antioxidant enzymes and by decreasing the expression of oxidative stress markers and inflammatory processes [83,84,85,86,87,88,89,90].

## 5. Conclusions

Topical formulation with natural ingredients is a new challenge and can lead to more safety and efficiency, in particular, if the used natural ingredients are a multifunctional material. We aimed in the present study to investigate the potential use of propolis as an aesthetic and a phytotherapeutic constituent of phytocosmetics. EEP of Algerian propolis could be used in such formulations and could act as a multifunctional constituent. The tested extract exhibited interesting antioxidant and antibacterial activities. Chemical analysis allowed the identification of 11 phenolics. Among them, five are considered the main compounds of the studied propolis, namely, chlorogenic acid, gallic acid, rutin, caffeic acid, and trans-cinnamic acid. In light of the present findings, propolis cannot only be used as a cosmetic ingredient but also be used as a preventative and curative constituent, which might be used as a barrier when applied externally on infected and non-infected skin.

## Figures and Tables

**Table 1:** MIC (Minimum Inhibitory Concentration) mg/mL of ethanolic extract of Algerian propolis.

Bacterial Strains	MIC (mg/mL)
**Standards strains**	
*S. aureus ATCC 25923*	0.06 ± 0.00 ^a^
*E. coli ATCC 25922*	1.00 ± 0.00 ^b^
*P. aeruginosa ATCC 27853*	1.10 ± 0.01 ^b,c^
**Clinical strains**	
*S. aureus*	0.20 ± 0.01 ^d^
*β hemolytic streptococcus*	0.60 ± 0.01 ^a^
*α hemolytic streptococcus*	>1.40 ^e^
non-hemolytic *streptococcus*	0.60 ± 0.01 ^a^
*E. coli*	1.00 ± 0.00 ^b^
*P. aeruginosa*	1.10 ± 0.01 ^b,c^
*P. mirabilis*	1.20 ± 0.02 ^c^
*K. pneumoniae*	1.20 ± 0.02 ^c^

MICs are expressed as mg/mL. Data are expressed as mean ± SD (*n =* 3). Values in the same column followed by the same lower-case letter are not significantly different.

**Table 2:** Macroscopic observation of healing process in experimental (treated) and control group.

Day of Receiving Treatment	Petroleum Jelly Untreated Rats	30% EEP	Propolis Ointment
1	Inflammatory reactions and a skin edema around the wound		
3		The wounds were smaller with a formed scab. Edema and reddening at the wound were visually smaller	
6	Wounds were covered with poorly formed scrab	Wounds were clean with a correctly developed scab	
9	No changes when compared to day 6	Wounds were covered with smaller scabs and on the skin border of the wound there was a pink scar	Dry wounds. Formation of dry scabs
18	Wounds covered with a light-pink epithelium	Wounds were healed completely. During palpation, the animals did not react defensively	

**Table 3:** UFLC/MS-MS analysis of phenolic acid composition of ethanolic extract of Algerian propolis EEP.

Compounds	Rt (min)	([M − H]^−^)	Concentration (ng /mL)
Gallic acid	7.9	169	44.25 ± 6.40
Chlorogenic acid	8.31	353	48.79 ± 5.01
*p*-hydroxybenzoic acid	8.36	137	ND
Rutin	8.43	609	21.12 ± 3.57
*p*-coumaric acid	8.69	163	ND
Caffeic acid	8.78	179	28.19 ± 4.95
Vanillic acid	8.8	167	4.24 ± 3.27
Syringic acid	8.86	197	7.69 ± 1.36
Sinapic acid	9.2	223	3.32 ± 2.61
Ferulic acid	9.34	193	11.48 ± 2.29
Trans cinnamic acid	10.2	147	20.10 ± 6.51

Data are presented as mean ± SD of ten parallel measurements (*n* = 10); Rt: retention time.

## Data Availability

The data presented in the present study are available within the article.
